# Quantitative identification of regular texture spacing via spatiotemporal signal analysis of pressure sensor responses

**DOI:** 10.3389/fnins.2026.1844208

**Published:** 2026-09-23

**Authors:** Wuyue Zhang, Bin Deng, Yanfei Jiao, Tian Gao, Tianshi Gao

**Affiliations:** 1 School of Electrical and Information Engineering, Tianjin University, Tianjin, China; 2 China Aerospace Science and Technology Corporation, China Academy of Aerospace System and Innovation, Beijing, China

**Keywords:** artificial neural network, attention mechanism, pattern recognition, tactile sensing, wearable devices

## Abstract

Tactile sensing in biological systems is a sophisticated spatiotemporal process where mechanoreceptors decode surface properties through rhythmic interactions. However, current artificial tactile recognition predominantly relies on discrete classification under highly constrained conditions, making it difficult to capture the quantitative physical dimensions of stimuli in naturalistic settings. This study proposes a spatiotemporal method for the quantitative identification of regular texture spacing under naturally varying tactile interaction conditions, bridging the gap between raw sensor dynamics and geometric perception. We utilized a prosthetic finger integrated with a piezoresistive sensor array to capture tactile responses during dynamic sliding. To characterize the frequency-tuning of the sensor array and the energy distribution of rhythmic excitations, Spectral Centroid Analysis (SCA) was employed for initial feature extraction, revealing a spatial coupling effect during tactile interaction. Subsequently, a hierarchical deep learning model was developed, combining Convolutional Neural Networks (CNN) to extract localized spatial contact patterns and Long Short-Term Memory (LSTM) units to integrate quasi-periodic temporal modulations. A channel-wise attention mechanism was incorporated to adaptively weight the contributions of non-uniform sensing regions. Experimental results demonstrate that the proposed approach achieves a recognition accuracy of 76.80% in identifying texture spacing, while indicating improved stability compared to baseline models that lack spatiotemporal integration. Crucially, interpretability analysis reveals that the attention mechanism effectively prioritizes informative sensing channels amidst the signal variability induced by unconstrained force. Our work establishes a attention-based approach to tactile decoding, demonstrating that the coupling of spatial heterogeneity and temporal periodicity is essential for robust perception. This approach provides a computational foundation for developing advanced neuro-prosthetics and autonomous robots capable of human-like tactile discernment in unconstrained environments.

## 1 Introduction

Tactile sensing is essential for humans to perceive the physical world, enabling the evaluation of object properties such as size (de Vignemont et al., 2005), shape (Hsiao, 2008), and temperature (Bergmann Tiest and Kappers, 2009). Beyond functional perception, it facilitates the development of bodily awareness and the neural discrimination between “self” and “non-self” (Dahiya et al., 2010). As a fundamental perceptual modality across biological systems (Handler and Ginty, 2021), biological tactile mechanisms have inspired the development of artificial electronic skins utilizing stretchable electronics (Yao et al., 2020) and flexible microfabricated sensors (Carluccio et al., 2023). In robotics and neuro-prosthetics, tactile feedback is indispensable for stable environmental interaction, especially when visual inputs are occluded or unreliable. Rather than a mere redundant modality, tactile sensing operates as a high-bandwidth spatiotemporal channel, allowing robot agents to actively infer surface properties through dynamic contact and oscillatory excitation (Schmitz et al., 2010; Koiva et al., 2013; Li et al., 2020).

In recent years, significant efforts have been directed toward tactile texture recognition, typically framed as a pattern classification problem. Conventional approaches utilize machine learning to decode vibration or pressure signals collected during sliding interactions. For instance, Huang and Wu (2021) implemented a biomimetic sensor to capture rhythmic vibrations, applying Support Vector Machines (SVM) and Random Forests to achieve high classification accuracies. With the advent of deep learning, Convolutional Neural Networks (CNN) and hybrid architectures have been increasingly adopted to automatically extract discriminative features from raw tactile time-series (Song et al., 2022). Recent advances have further introduced spatio-temporal attention models to capture the intricate correlations between the spatial arrangement of the sensor array and the temporal evolution of contact sequences, enhancing performance across large-scale material datasets (Cao et al., 2020).

However, a critical gap remains in our understanding of how artificial intelligence systems encode the underlying physical dimensions of a stimulus. Current research predominantly treats tactile perception as a discrete “black box” classification task, often marginalizing the computational principles by which rhythmic mechanical excitations are transformed into quantitative physical representations. Furthermore, many existing paradigms rely on highly controlled experimental conditions, such as maintaining a strictly constant pressing force or a fixed contact trajectory to minimize signal variance. This approach differs from naturalistic interactions in daily life, where biological or robotic hands engage with objects through unconstrained and dynamic movements. In such real-world scenarios, the interaction is characterized by fluctuating contact forces and varying scanning velocities, which introduce significant non-stationary noise and amplitude modulations into the tactile stream. In biological systems, mechanoreceptors such as Meissner and Pacinian corpuscles perform a form of frequency tuning (Gao T. et al., 2025), where the temporal structure of the neural spike train is intrinsically linked to the spatial periodicity of the texture (Saal and Bensmaia, 2014). Specifically, few studies bridge the gap between measurable surface geometries, such as ridge spacing, and the emergent spatiotemporal signal patterns in a way that allows for quantitative identification rather than mere qualitative labeling. Elucidating this relationship is essential, as surface microstructures govern the mechanical energetics that shape the perceptual input. Consequently, it remains a question how geometric parameters, notably texture spacing, are dynamically encoded within the high-dimensional spatiotemporal flow under the inherent variability and unconstrained dynamics of natural physical interaction.

To address this, we design an approach that explicitly treats tactile responses as attention-based spatiotemporal signals. When a sensor slides over periodic ridges with spacing *d* at a velocity *v*, it generates a quasi-periodic excitation with a fundamental frequency *f*≈*v*/*d*. This process exhibits three intrinsic characteristics that a robust decoder must account for: (1) spatial heterogeneity across the afferent sensing array; (2) quasi-periodic temporal modulation governed by the texture's spatial frequency; and (3) non-stationary amplitude variations induced by unconstrained tactile interaction. Consequently, in this study, we develop a hierarchical spatiotemporal approach for texture spacing discrimination, aiming to mirror the ascending stages of tactile information processing. Using a prosthetic finger equipped with a piezoresistive sensor array, we analyze the frequency-domain characteristics via Spectral Centroid Analysis (SCA). Subsequently, we employ convolutional layers to capture localized spatial interaction patterns, followed by Long Short-Term Memory (LSTM) units to integrate the quasi-periodic temporal dynamics (Lee et al., 2017). To account for the non-uniform importance of different sensing regions, a channel-wise attention mechanism is introduced. The main contributions of this work are summarized as follows:

1. We quantitatively characterize regular texture spacing through spatiotemporal and spectral analysis of tactile responses, with Spatial statistical analysis and spectral centroid mapping revealing non-uniform sensing patterns across the pressure sensor array;
2. We design an attention-based decoding approach that integrates spatial convolution, temporal memory, and an adaptive weighting strategy to robustly capture tactile dynamics under unconstrained pressing forces;
3. We provide an interpretable analysis of channel-wise contributions, revealing how spatially non-uniform sensing regions contribute to the computational encoding of periodic structural information.

It should be emphasized that the objective of this study is not to establish a new benchmark for large-scale classification, but to investigate the encoding mechanism of periodic structural spacing within tactile dynamics. The proposed approach serves as a computational tool to analyze this encoding process, providing insights into how physical surface properties are manifested in the spatiotemporal responses of artificial tactile systems.

## 2 Materials and methods

### 2.1 Dataset acquisition

To obtain editable, structured data, we established a pipeline from analog inputs via a tactile pressure sensor array to digital outputs packed by the terminal; its data flow is shown in Figure 1.

**Figure 1:**
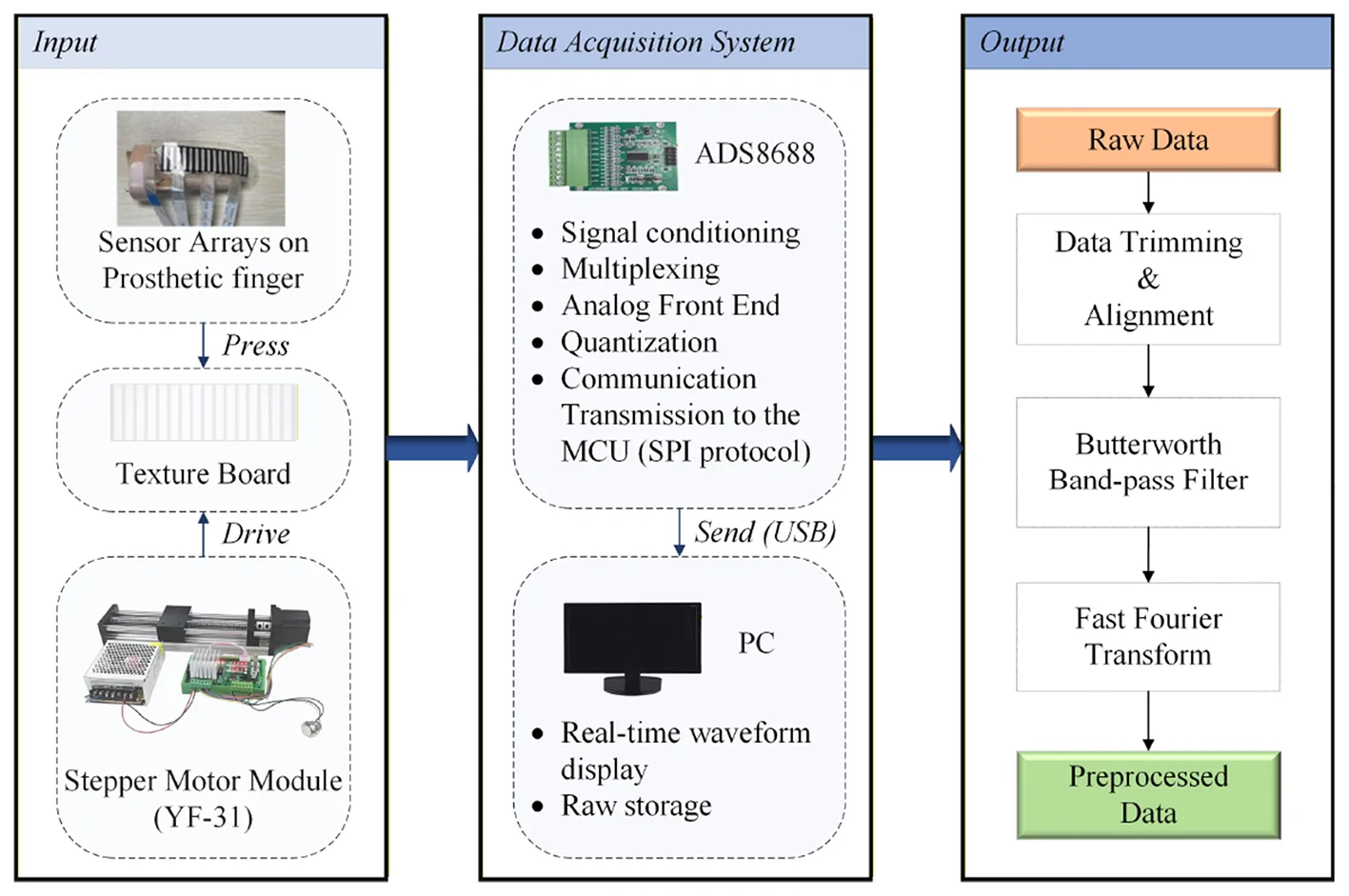
Block diagram of tactile signals data flow.

#### 2.1.1 Experimental protocol

A single 15 × 4 flexible pressure sensor array (pertinent parameters listed in Table 1) was affixed to the prosthetic index finger. This data acquisition device was designed based on the piezoresistive effect. When the sensor is subjected to pressure, the resistance decreases as the pressure increases. As shown in Figure 2, its piezoresistive characteristics indicate that resistance and pressure are in a power function relationship, and the inverse of resistance is in an approximately linear relationship with pressure.

**Table 1:** Parameters of the stepper motor.

Variable	Value
Model	57HS56-3004-001
Step angle	1.8°
Phase current	3.0 A
Phase resistance	1.0 Ω
Phase inductance	2.9 mH
Static torque	13 kg.cm
Moment of inertia	300 g·cm^2^

**Figure 2:**
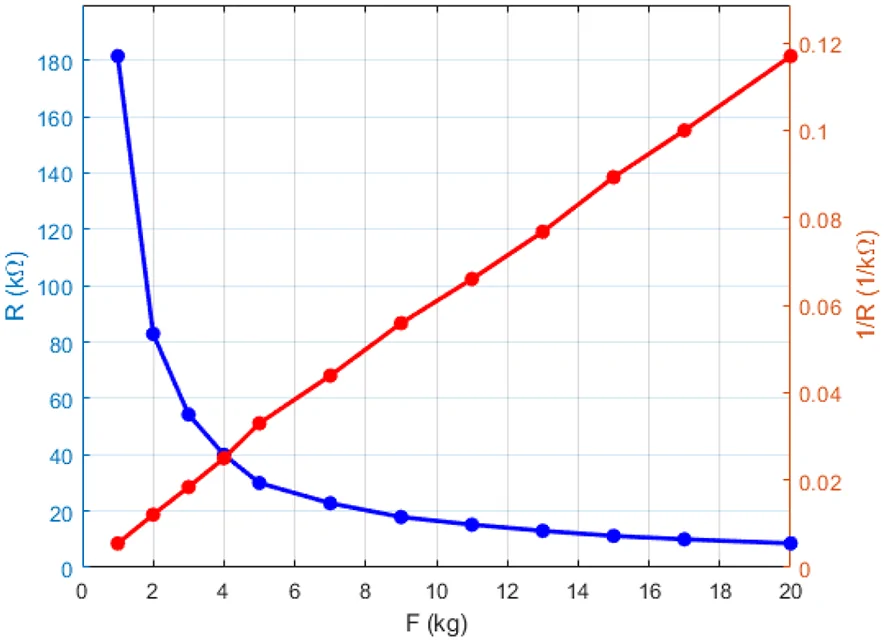
The corresponding relationship between force and resistance and its derivative. The relationship between force and conductivity is approximately in direct proportion.

During the experiments, the subjects wore this finger and performed natural pressing interactions with unconstrained contact force onto a texture board with regular spacing, while a YF-31 hybrid stepper motor driver (pertinent parameters are listed in Table 2) dragged the texture board at a constant speed to achieve relatively stable linear movement between the fingertip and the texture board. The pulse frequency of the stepping motor was set to 9,600 Hz, the speed was 360 RPM (actual error ±4%), and a constant sliding velocity (*v* = 30 mm/s) was maintained throughout the trials. During experiments, an acquisition card (Figure 3a) continuously received and uploaded raw tactile data to a terminal. All participants were informed of the experimental procedures prior to participation and provided informed consent. The study involved two healthy adult male volunteers performing non-invasive tactile perception experiments using a prosthetic finger attachment. The experimental procedures involved minimal risk and did not include any medical intervention, biological sampling, or collection of personally identifiable information. The study was conducted in accordance with the ethical principles of the Declaration of Helsinki.

**Table 2:** Training hyperparameters and implementation settings.

Parameter	Value	Description
Epochs	200	Number of training epochs
Optimizer	Adam	Optimization algorithm
Learning rate	0.002	Initial optimizer learning rate
Batch size	4	Samples per mini-batch
Kernel size	2	Convolution kernel size
Hidden dimension	64	Hidden size of the LSTM layer
Random seed	42	Seed for reproducibility
Loss function	Cross-Entropy	Training objective

**Figure 3:**
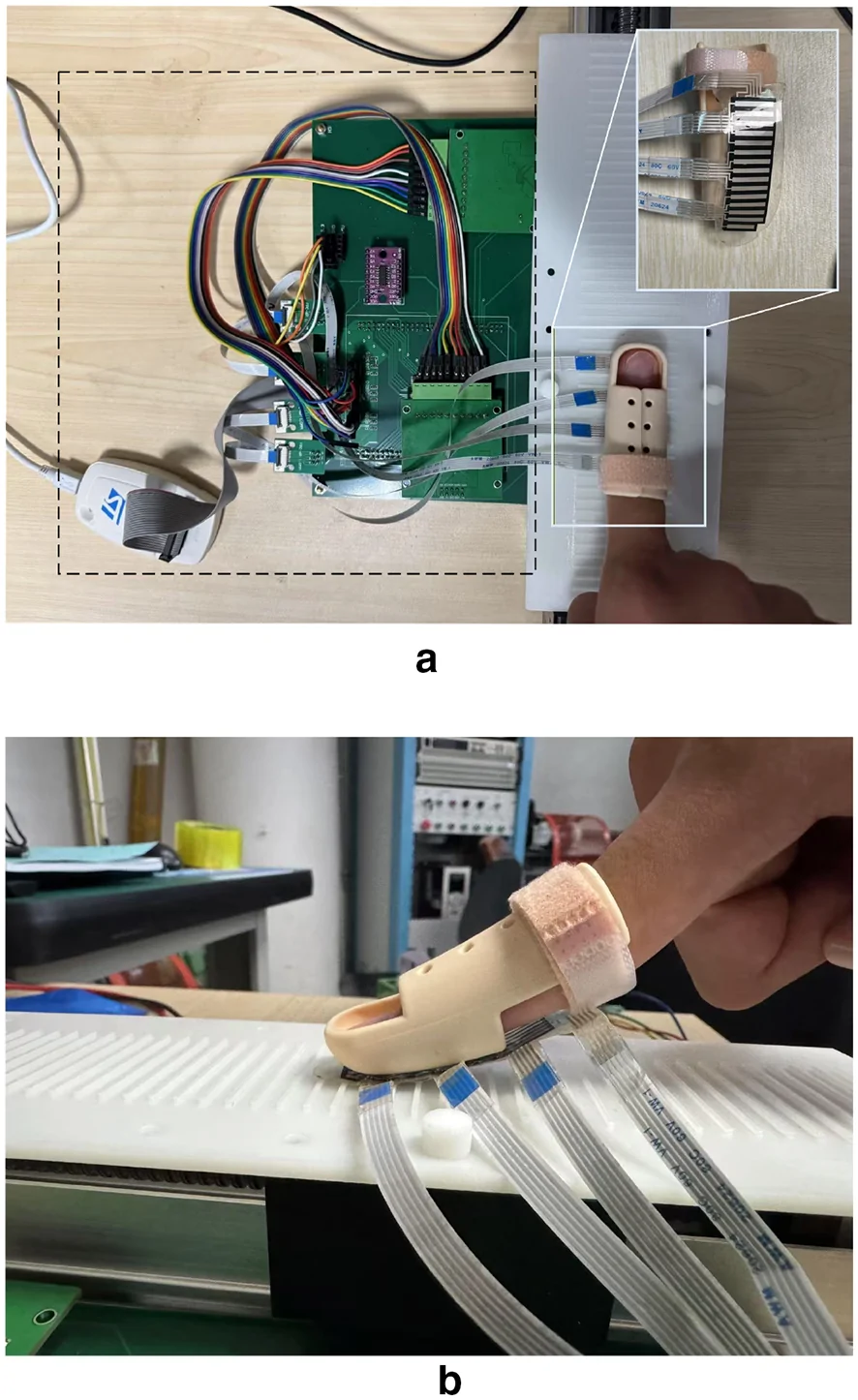
Schematic diagram of the experimental process. **(a)** The subjects wore a prosthetic finger and pressed the texture board. The data collected by the experiment was received by the acquisition card (in the black dotted box) and uploaded to the PC. **(b)** A side view of the experimental process. The subjects were asked to stay in contact with the texture board during experiments.

Crucially, the applied contact force was not strictly constrained to be constant across different texture spacing conditions. Instead, the experimental protocol was devised to allow unconstrained pressing behavior (Figure 3b), thereby enabling the pressure sensors to capture the variations in pressure naturally applied by the subjects.

This intentional design acknowledges that practical robotic or prosthetic applications rarely encounter perfectly constant contact forces; tactile perception systems must operate under non-ideal, dynamically varying interaction conditions. Consequently, this study aimed to investigate whether spatiotemporal signal characteristics related to texture spacing remain discriminative despite realistic force fluctuations.

#### 2.1.2 Dataset specification

The length of the texture board is 25 cm. Small cuboids with a cross-sectional area of 1 mm × 1 mm form the texture pattern, ensuring that the texture presents a regular arrangement of ridges and grooves (Figure 4a). The spacing *d* between these cuboids ranged from 3 to 7 mm (step 1 mm), and corresponding multiple sets of repeated experiments (10 times per texture condition) were carried out (Figure 4b). It is noted that the use of regularly spaced textures is intentional rather than restrictive, as such structures generate quasi-periodic tactile excitation with a well-defined fundamental frequency determined by the relation *f* = *v*/*d*, where *v* is the sliding velocity and *d* is the spacing. They are also close to the tactile resolution of the human hand (Van Boven and Johnson, 1994). This approach facilitated a systematic investigation into how spacing information is encoded in spatiotemporal tactile signals under specific texture conditions.

**Figure 4:**
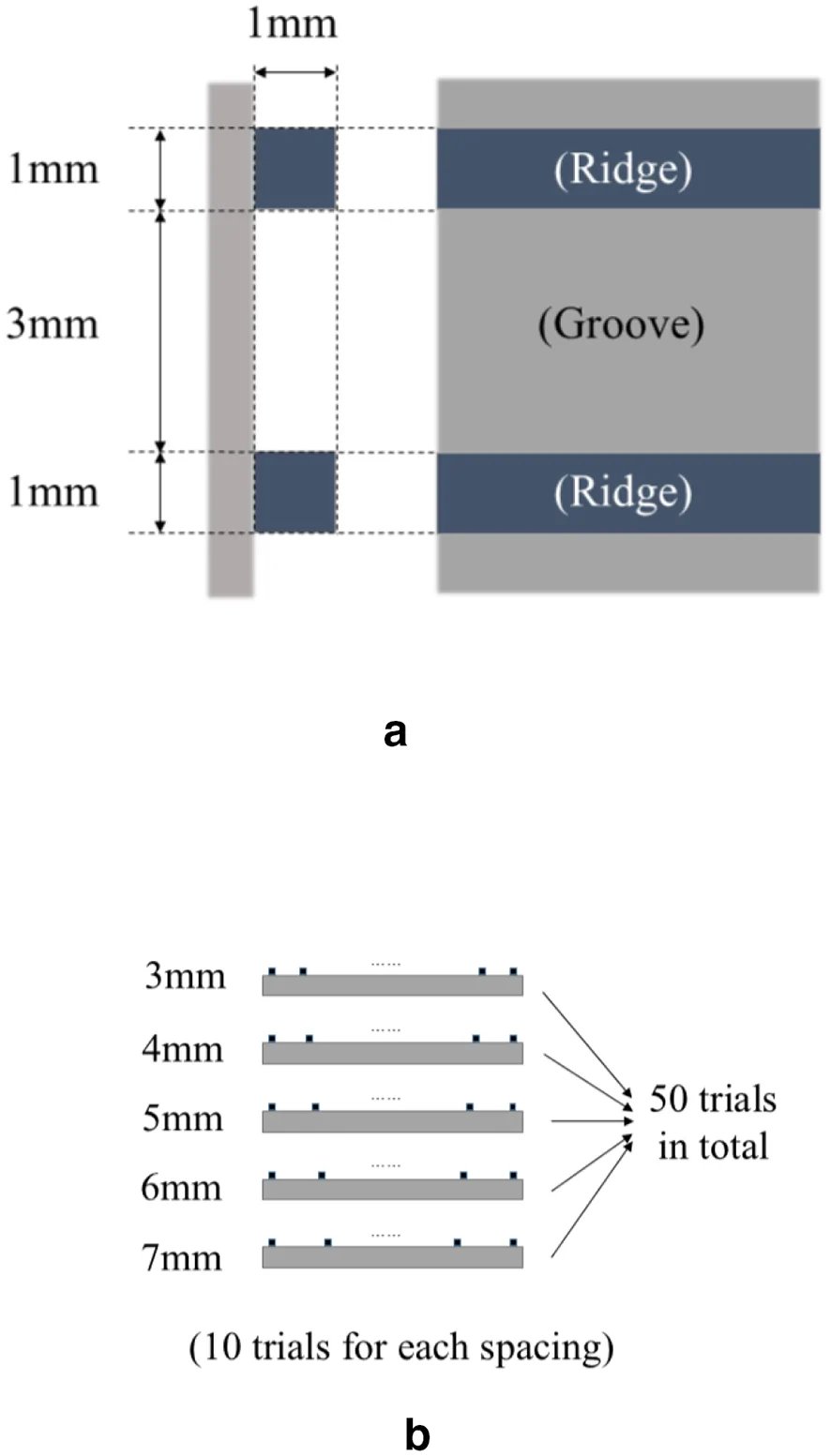
Texture details and the composition of the dataset. **(a)** Details the geometric dimensions of the ridges and grooves (taking 3 mm as the example), which determine the frequency response of the input signal. **(b)** The dataset distribution for five different spatial frequencies (3–7 mm spacing).

#### 2.1.3 Data preprocessing

Before formally analyzing the tactile sensing data, the experimental data collected by the acquisition system need to be preprocessed. Since the subject was only required to press the texture board with the distal phalanx of the index finger, and only the first 40 sensing units were adhered to the fingertip, the signals received from these 40 sensor units (10 × 4) were considered valid. After normalization and synchronization with the experimental timeline, the initial contact phase (finger pressing onto the textured surface) and the final detachment phase were removed to retain only the steady sliding interaction segment for analysis. Given the motor-driven sliding velocity *v* = 30 mm/s and the texture spacing *d* ranging from 3 to 7 mm, the fundamental tactile excitation frequency can be approximated by *f*_exc_ = *v*/*d*, which yields a frequency range of approximately 4.3–10 Hz. Although the primary modulation frequency lies within this band, higher harmonics and transient components generated by non-linear contact interactions may extend beyond the fundamental frequency.

The signals were sampled at 385 Hz (2.6 ms interval), which satisfies the Nyquist criterion for the expected excitation band. Specifically, this sampling rate provides a Nyquist frequency of 192.5 Hz, which strategically covers the sensitive range of Fast-Adapting type I (FA-I) units–the biological counterparts responsible for detecting low-frequency vibrations and micro-slips during texture interaction (Abraira and Ginty, 2013). The corresponding frequency resolution is Δ*f* = *f*_*s*_/*N*, where *f*_*s*_ denotes the sampling frequency and *N* the number of samples within each analysis window. Therefore, this sampling rate provides adequate spectral resolution while maintaining a manageable data volume and computational efficiency.

To suppress low-frequency drift and high-frequency noise, a fourth-order Butterworth band-pass filter with cut-off frequencies of 1 and 100 Hz was applied. The lower cut-off removes quasi-static baseline drift, whereas the upper cut-off attenuates high-frequency electronic noise and mechanical vibrations outside the relevant tactile response band. Before model training, the tactile signals from each sensing channel were normalized to [0,1] using min-max normalization independently to reduce inter-channel amplitude differences while preserving the temporal characteristics of the signals.

### 2.2 Spectral feature extraction

In order to explore the frequency domain characteristics of the signal received by each sensing unit, the discrete Fourier transform (DFT) was first computed using the Fast Fourier Transform (FFT) algorithm to obtain the frequency domain representation of the steady-state vibration signals.

To further quantitatively characterize the frequency distribution of the vibration signals, SCA was applied in the frequency domain (Tang et al., 2015). For each sensing unit *k*, the time-domain signal *s*_*k*_(*t*) was transformed into its discrete frequency-domain representation using DFT, yielding the complex spectrum *S*_*k*_(*f*_*m*_), where *f*_*m*_ denotes the *m*-th discrete frequency bin. The spectral centroid for sensing unit *k* is defined as


$$\begin{array}{c} SC_{k}=\frac{\sum_{m}f_{m}|S_{k}\left(f_{m}\right){|}^{2}}{\sum_{m}|S_{k}\left(f_{m}\right){|}^{2}}, \end{array}$$ (1)

where *f*_*m*_ represents the discrete frequency values corresponding to the DFT bins, $|S_{k}\left(f_{m}\right){|}^{2}$ denotes the power spectrum of the signal, and *m* indexes the frequency bins. Equation [1] thus reflects the center of gravity of the power spectral distribution and provides a quantitative descriptor of the dominant frequency components of the vibration signal.

### 2.3 Network architecture

In this study, the tactile input at each time step can be represented as a spatial pressure distribution across the sensor array. Since adjacent sensing units often exhibit correlated responses due to local contact geometry, convolutional operations are employed to extract localized spatial interaction patterns. Unlike fully connected mappings, convolutional kernels (Krichen, 2023) preserve spatial adjacency information and enable the model to learn structured pressure variations associated with texture ridges and grooves (Gandarias et al., 2019; Jie and Wanda, 2020).

Meanwhile, the temporal sequence processed by the LSTM corresponds to successive spatial pressure maps acquired during sliding interactions. The periodic excitation induced by texture spacing results in repeated but amplitude-modulated temporal patterns. The gating mechanism of the LSTM allows the network to selectively retain frequency-consistent components across multiple excitation cycles while suppressing transient fluctuations caused by force variability (Hochreiter and Schmidhuber, 1997). Thus, the LSTM layer serves as a temporal integrator of quasi-periodic tactile dynamics rather than a generic sequence processor.

From a tactile sensing perspective, tactile sensing units contribute disproportionately to texture discrimination. Inherent variability in local curvature, contact pressure, and sliding alignment induces non-uniform periodic modulation across the sensor array. To optimize information extraction, an attention mechanism can be integrated to adaptively reweight sensing units (Ma and Mei, 2022; Wang et al., 2024). Specifically, the Squeeze-and-Excitation (SE) block recalibrates channel-wise feature responses by modeling interdependencies through sequential “Squeeze” and “Excitation” operations (Hu et al., 2018):

1. Squeeze operation: global average pooling is applied along the temporal dimension to aggregate channel-wise information, producing a compact channel descriptor of size 1 × C, where C represents the number of channels;
2. Excitation operation: the channel descriptor is passed through a non-linear gating mechanism with a sigmoid activation to generate adaptive channel weights.

The SE mechanism enables adaptive channel-wise weighting, effectively modeling the non-uniform spatial contribution of different sensing units. This is particularly important under unconstrained force conditions, where discriminative information may concentrate in specific signal channels (Deng et al., 2023). When embedded into ResNet, the resulting ResNet-SE structure significantly improves feature representation capability and classification accuracy with only a minor increase in computational cost (Hu et al., 2018). The overall architecture is shown in Figure 5a.

**Figure 5:**
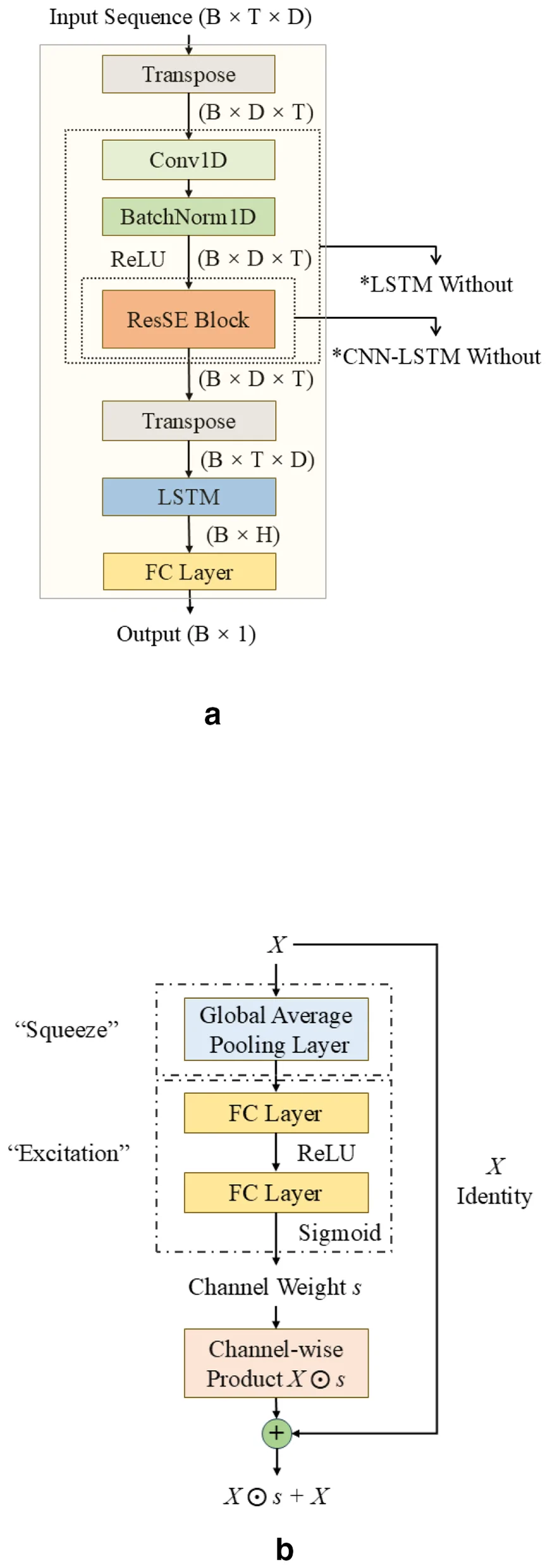
Architecture of the proposed ResSE-CNN-LSTM model. **(a)** The overall structure of ResSE-CNN-LSTM model, where *B* represents the batch size, *D* represents the dimensionality of signals, *T* represents the length, *H* represents the hidden size of LSTM. **(b)** The detailed structure of the ResSE block.

### 2.4 Implementation details

To enhance model versatility across variable-length tactile sequences and augment sparse datasets, we implemented three augmentation techniques:

1. Variable-length window slicing: randomly cropping segments (250–2,250 points) from arbitrary starting indices to facilitate multi-scale temporal processing;
2. Gaussian noise: injecting channel-wise noise (σ = 0.1) to simulate sensor measurement errors and bolster robustness;
3. Time jitter: temporal rescaling (factor of 20) to mimic sliding speed variations, promoting time-invariant feature extraction.

Through these data augmentations, the models were expected to maintain stable convergence despite a wide diversity of training samples and demonstrate strong generalization capabilities to data of varying lengths and slight noise (Gao Z. et al., 2025). To ensure the statistical significance and generalization of the results, given the relatively small sample size (50 original trials across five texture categories), a 10-fold stratified cross-validation strategy was implemented. In each fold, the Stratified K-Fold algorithm was utilized to partition the data, ensuring that the ratio of the five texture classes (3–7 mm spacings) in the training and testing sets remained consistent with the original distribution. To mitigate overfitting and simulate the stochastic nature of real-world tactile sensing, a dynamic sliding window augmentation mechanism was developed. Each original training trial was expanded by a factor of 20. The testing trials were augmented by a factor of 5 to provide a more comprehensive evaluation of the model's performance across different segments of the signal. To prevent information leakage, the original tactile recordings were first partitioned into training and testing sets at the trial level using stratified 10-fold cross-validation. Sliding-window augmentation was then performed independently within each subset. Therefore, all augmented samples in the testing set originated exclusively from testing trials, while training samples were generated only from training trials. No augmented segments derived from the same original trial were shared between the two subsets.

The experiments were conducted on a Windows workstation equipped with an AMD Ryzen 9 5900HX CPU and an NVIDIA GeForce RTX 3070 GPU. All models were implemented in PyTorch and trained using the Adam optimizer with an initial learning rate of 0.002. The other training hyperparameters and implementation settings are presented in Table 3.

**Table 3:** Comparisons among baseline models.

Model	LSTM layer	CNN block	ResSE block	Accuracy (%)	*F*1_macro_
LSTM	✓	×	×	65.60 ± 14.99	0.6600
CNN-LSTM	✓	✓	×	73.20 ± 5.67	0.7296
**ResSE-CNN-LSTM (Ours)**	✓	✓	✓	**76.80** **±5.31**	**0.7640**

Bold values stress the best performance of our proposed model. *F*1_macro_ is calculated based on Equations 2, 3.

## 3 Results

### 3.1 Frequency peak analysis of texture-dependent encoding

The dominant frequency analysis provides further evidence that texture spacing information is encoded in the temporal frequency domain. As shown in Figure 6a, the dominant frequency gradually decreases with increasing texture spacing, following the expected inverse relationship between spacing and excitation frequency (*f* = *v*/*d*). Although deviations exist between the measured peak frequencies and theoretical values, these differences are expected due to the non-linear contact dynamics, sensor bandwidth, and distributed tactile responses during sliding interactions.

**Figure 6:**
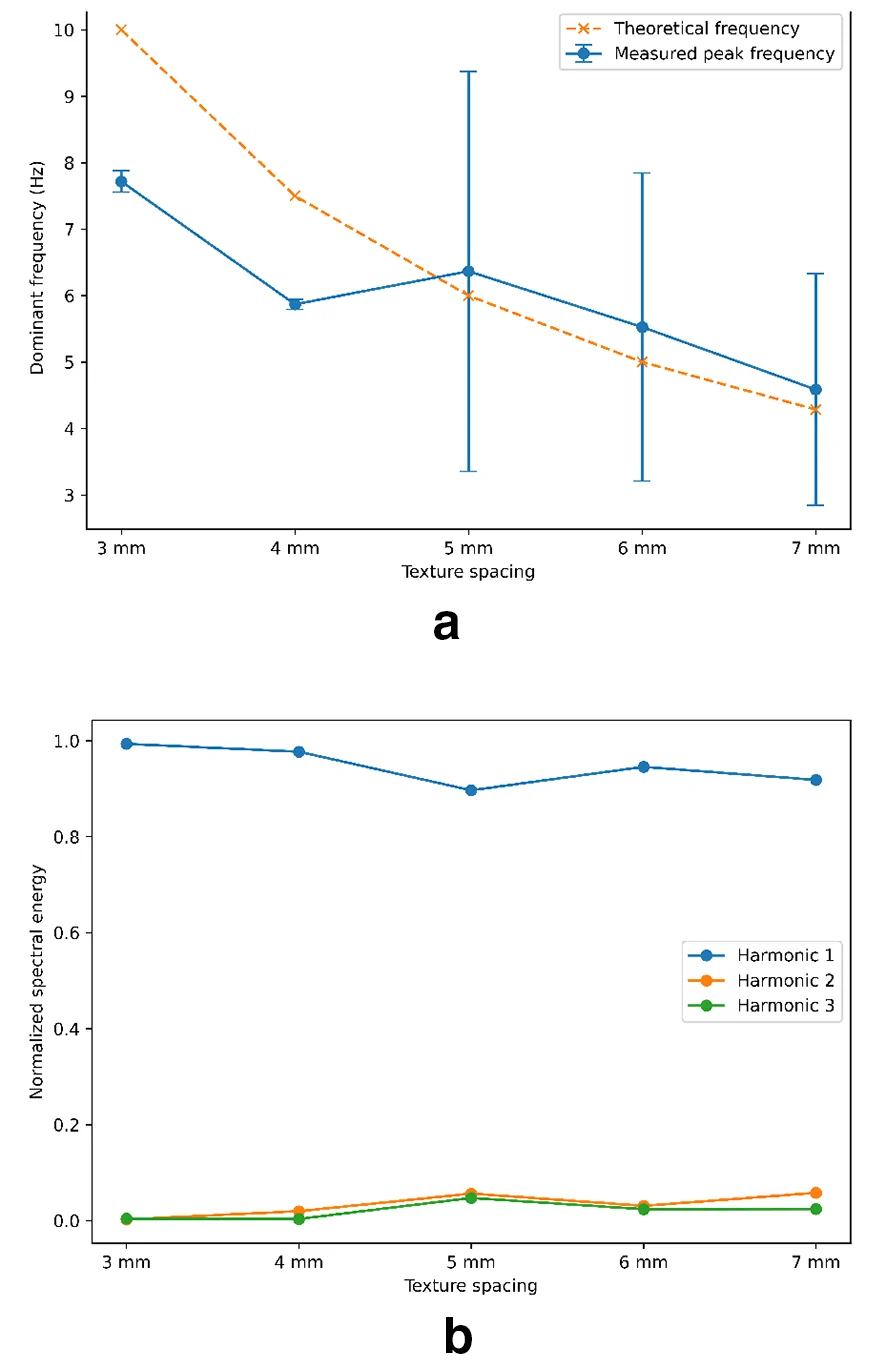
Frequency characteristics of the experimental tactile data. **(a)** The dominant frequency tracking. **(b)** The harmonic component analysis.

In addition to the dominant frequency analysis, harmonic components were further examined to characterize potential non-linear responses during tactile interaction. As shown in Figure 6b, the fundamental component accounted for the majority of spectral energy across all texture conditions, while the second-order and third-order harmonics exhibited relatively low energy contributions. This indicates that texture-dependent temporal encoding is primarily represented by the fundamental excitation frequency, with higher-order harmonics providing additional but limited spectral information.

### 3.2 Spatial heterogeneity in tactile interaction and spectral response

As shown in Figure 7, the results of SCA revealed that the dominant frequency of the signal did not follow the expected monotonic decrease with increasing texture spacing: the spectral centroid was higher in the 3 mm spacing group, decreased significantly at 4 mm, and rebounded slightly in the 5–6 mm spacing group before decreasing again at 7 mm. This phenomenon suggests that the spectral characteristics of tactile signals are affected not only by texture spacing but also by the complex interaction between the finger and the surface.

**Figure 7:**
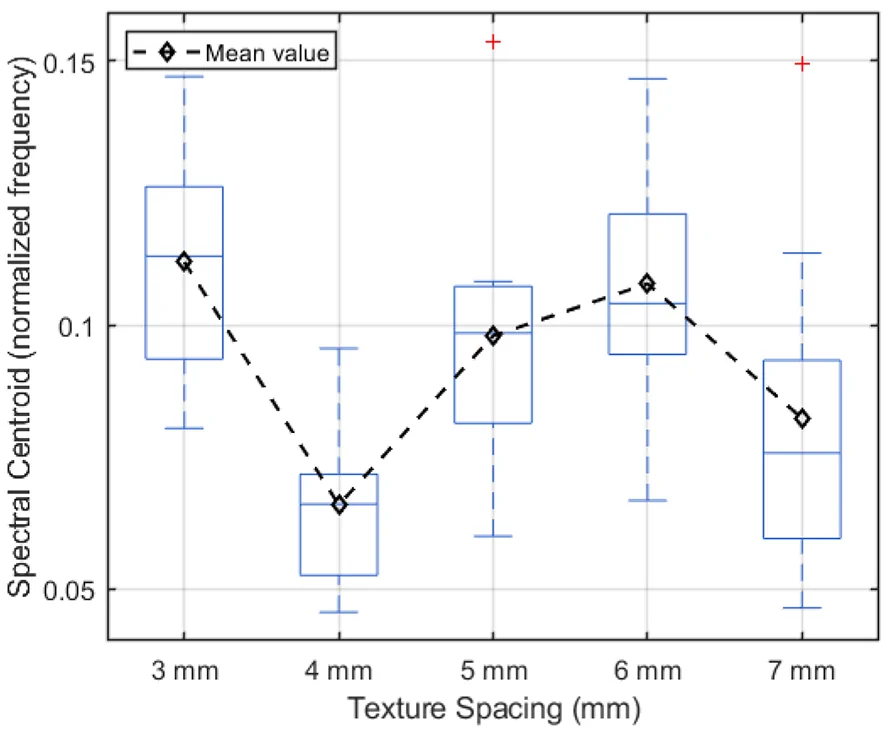
SCA results across texture spacing.

To further explore the spatial distribution characteristics of tactile signals, a one-way analysis of variance (ANOVA) was conducted on the spectral centroid of the 10 × 4 pressure sensor array. This method compares the ratio of the between-group variance to the within-group variance (i.e., the *F* statistic) to determine whether the distributions across different categories differ statistically significantly (Fisher, 1992; Montgomery, 2017). When this ratio is significantly above the level of random fluctuation, the feature is considered discriminative. ANOVA on spectral centroid across channels returned *F* = 17.2827, *p* < 0.05, indicating significant inter-channel differences. This finding suggests that even when the texture ridges and grooves are distributed at a fixed spacing, and the texture board moves uniformly, the contact between the finger and the texture board is not completely uniform when the subjects naturally press the texture board.

To visualize this region effect, spatial interpolation analysis of the spectral centroids for each texture spacing group was performed, and corresponding smoothed heatmaps were generated. The interpolated heatmaps display the distribution and gradient of the spectral centroids across the sensor units' space, further revealing the spatial characteristics of the local high-frequency response at different texture spacings, as shown in Figure 8. The observed spatial gradients in spectral centroid mirror the non-uniform energy distribution of the sensor array in the artificial fingertip, further confirming that tactile perception is inherently a spatiotemporal integration process rather than a static frequency matching task. This analysis not only supplements the trend information obtained from the average spectral centroids but also provides intuitive spatial evidence for understanding the interaction between the tactile sensing of the prosthetic finger and the texture board. It also indicates that there is a significant spatial coupling effect during the tactile signal acquisition process, and traditional frequency domain analysis methods cannot intuitively distinguish the texture spacing with a resolution of 1 mm, calling for high-performance algorithms for spatiotemporal information coupling processing as a solution.

**Figure 8:**
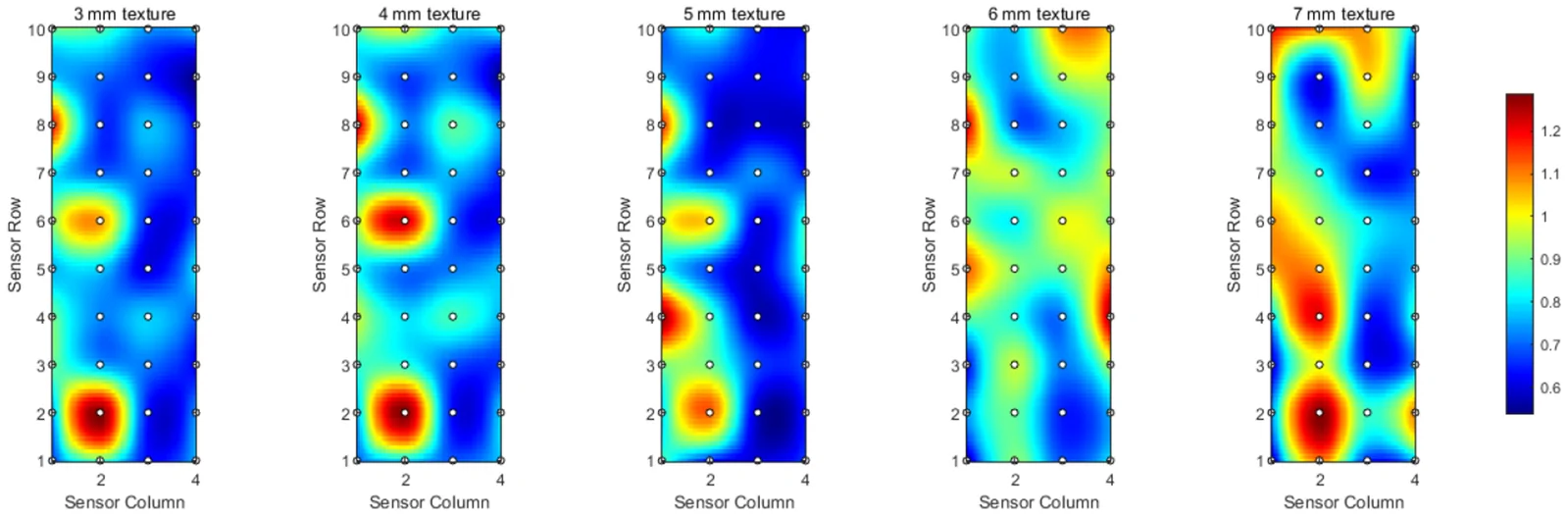
Spatial distribution of spectral centroid under different texture spacing (interpolated heatmaps).

### 3.3 Model evaluation and ablation analysis of ResSE-CNN-LSTM

For a multi-class classification problem with *N* categories, the macro-averaged F1-score is calculated by independently computing the F1-score for each category and then taking the arithmetic mean. The formula is given as follows:


$$\begin{array}{c} F1_{i}=\frac{1}{N}\overset{N}{\underset{i=1}{\sum }}\frac{2\times P_{i}\times R_{i}}{P_{i}+R_{i}} \end{array}$$ (2)

$$\begin{array}{c} F1_{\text{macro}}=\frac{1}{N}\overset{N}{\underset{i=1}{\sum }}F1_{i} \end{array}$$ (3)

where *P*_*i*_ and *R*_*i*_ represent the precision and recall for the *i*-th tactile texture category, respectively. *N* denotes the total number of categories (in this study, *N* = 5, corresponding to the 3, 4, 5, 6, and 7 mm texture spacing). The macro-averaging method ensures that each texture category contributes equally to the final metric, reflecting the model's robustness across all resolution scales.

To verify the contribution of each module in our proposed ResSE-CNN-LSTM architecture, we conducted an ablation study comparing the baseline LSTM, the CNN-LSTM, and the final ResSE-CNN-LSTM:

1. LSTM: a traditional LSTM model with a single layer and a single direction;
2. CNN-LSTM: an extra convolutional layer was added based on the traditional LSTM mentioned above;
3. ResSE-CNN-LSTM: based on the residual structure shown in Figure 5b, a ResSE block containing one global average pooling layer for the “squeeze” operation and one sequence of fully connected layers (FC layers) for the “excitation” operation was added based on the CNN-LSTM mentioned above;

The 10-fold cross-validation results demonstrate a progressive improvement in classification accuracy. As shown in Table 3, the baseline LSTM achieved a mean accuracy of 65.60% ± 14.99%. By introducing CNN layers for spatial-temporal feature extraction, the CNN-LSTM improved the accuracy to 73.20% ± 5.67%, highlighting the importance of convolutional filters in capturing local tactile patterns. Finally, our ResSE-CNN-LSTM achieved the highest performance of 76.80% ± 5.31%. This accuracy improvement and reduction in standard deviation over CNN-LSTM confirm that the residual-structure-based SE block refines the feature maps by emphasizing informative channels and suppressing noise, leading to a more robust classification of texture spacings.

### 3.4 Multi-model comparison and interpretability of attention mechanism

We compare our ResSE-CNN-LSTM with several classical and state-of-the-art models, including SVM, GFF-PDCN, and Deep-CNN-LSTM. The final results are summarized in terms of Mean Accuracy ± Standard Deviation, as shown in Table 4.

**Table 4:** Comparisons among different network structures.

Model	Accuracy (%)	*F*1_macro_
GFF-PDCN (Zhu et al., 2026)	27.20 ± 14.40	0.2622
SVM (Suykens, 2001)	73.60 ± 12.16	0.7363
Deep-CNN-LSTM (Awan et al., 2023)	75.60 ± 7.03	0.7554
**ResSE-CNN-LSTM (Ours)**	**76.80** **±5.31**	**0.7640**

Bold values stress the best performance of our proposed model. *F*1_macro_ is calculated based on Equations 2, 3.

As indicated in the 10-fold results, our model (76.80% ± 5.31%) show improved performance compared with SVM (73.60% ± 12.16%) and Deep-CNN-LSTM (75.60% ± 7.03%). Notably, the GFF-PDCN model performed poorly on this dataset, with an accuracy of only 27.20% ± 14.40%, suggesting it struggled to converge on the specific tactile features provided.

The statistical significance of the performance gains was evaluated using *p*-values. Our ResSE-CNN-LSTM model shows a statistically significant improvement over the GFF-PDCN (*p* < 0.001). While the accuracy improvement over SVM (73.60%–76.80%) and Deep-CNN-LSTM (75.60%–76.80%) did not reach the conventional 0.05 threshold (*p* = 0.479 and *p* = 0.604, respectively), the ResSE-CNN-LSTM consistently exhibited the lowest standard deviation (5.31%), indicating superior stability and reliability in tactile recognition across different data splits.

The normalized confusion matrix (Figure 9) shows that the model achieves high accuracies for the 7 mm (0.96) and 3 mm (0.88) categories. The main classification challenge occurs between the 5 and 6 mm samples, where 32% of the 5 mm ground truth samples are misclassified as 6 mm, which may be related to the coupling effect with the stepper motor operating frequency (6 Hz). Furthermore, the interpretability of our model is supported by the evolution of SE channel attention weights. As shown in Figure 10, over 50 training epochs, the weights of critical feature channels (e.g., Channel 6–21) converge toward 1.0, while redundant or noisy channels (e.g., Channel 38–39) drop toward 0.0, demonstrating the model's ability to autonomously learn and focus on the most important channels.

**Figure 9:**
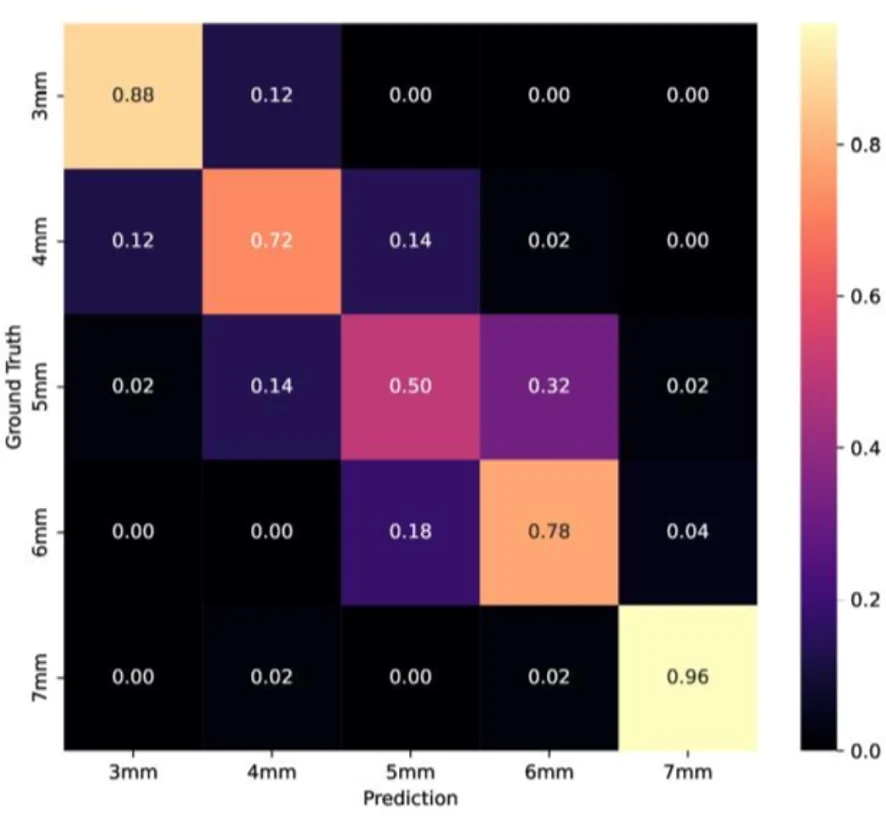
Confusion matrix (ResSE-CNN-LSTM).

**Figure 10:**
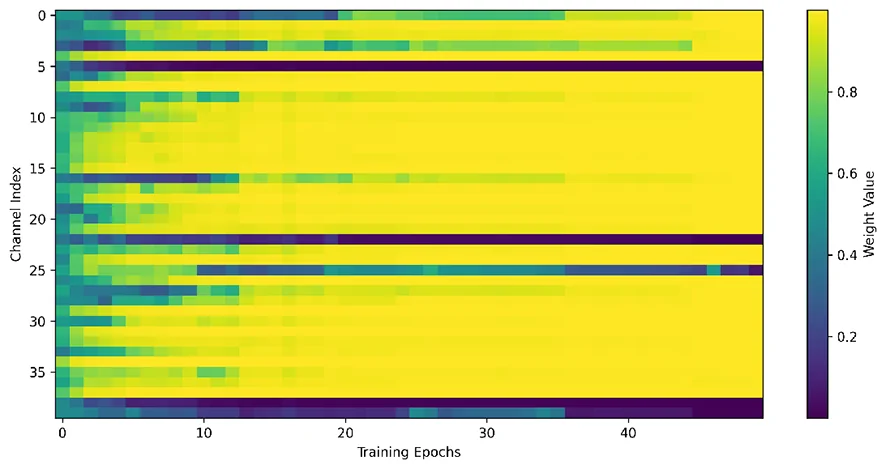
The heatmap of weight evolution during training.

Furthermore, this channel-wise attention mechanism explains why the ResSE-CNN-LSTM maintains the lowest standard deviation (5.31%) among all tested architectures. By autonomously de-emphasizing redundant dimensions, the model prevents the overfitting typically associated with high-dimensional tactile data, ensuring that the decision boundary is governed by stable tactile features relevant to the physical environment rather than stochastic noise. This interpretability validates the effectiveness of the Squeeze-and-Excitation block for adaptive channel weighting in tactile sensing.

## 4 Discussion

In tactile texture recognition tasks, aligning the mechanical excitation frequency with the dominant frequency band of the tactile signal is not merely for sampling convenience (Wettels et al., 2008); it stems from engineering considerations regarding signal excitation efficiency and information separability. If the driving frequency significantly mismatches the inherent tactile frequency band induced by the texture, the sensor may not be sufficiently excited within the target frequency band, resulting in insufficient effective signal energy and a reduced signal-to-noise ratio (SNR). Conversely, if the driving frequency is too high or too low, the texture feature distribution may be compressed to the spectral edges or even destroyed during preprocessing filtering, leading to inter-class feature blurring. Therefore, in this study, the motor rotation frequency was set to 6 Hz. By achieving frequency-domain energy coupling with the target texture frequency band (*f* = *v*/*d* controlled within the range of 4.3–10 Hz), the mechanical vibrations induced by the texture can be most effectively mapped to the sensor's frequency band, thereby improving sensing efficiency. Furthermore, the pressure-sensitive tactile sensors used have a response time of less than 20 ms (as shown in Table 5), which means they can fully cover signal variations within the frequency range *f* and prevent sampling or response delays from affecting data quality. However, the utilization rate of the sensor array in this experiment was relatively low (approximately 66%), and the finger-shaped tactile patches constructed from the array are only suitable for fingers of limited size. Reconstruction of tactile array is a promising potential solution (Legrand et al., 2023).

**Table 5:** Parameters of the pressure sensor unit.

Variable	Value
Model	RX-M1616S
Range	1–50 kg
Hysteresis	< 6%
Drift	< 8%
Operating voltage	3.3–5 V
Operating temperature	−50 to +50 °C
Response time	< 20 ms
Service life	>500,000 times

The results of the SCA underscore that tactile perception of texture spacing is not a simple linear mapping of periodic excitations. The observed non-monotonic shift in spectral centroids across 3–7 mm spacings suggests that the interaction between the prosthetic finger and the textured surface is governed by complex contact mechanics rather than idealized frequency responses. Unlike theoretical simulations, naturalistic pressing involves significant spatial heterogeneity, as evidenced by the high *F*-statistic in our ANOVA results. This spatial non-uniformity implies that SCA provides only macroscopic information about the frequency-domain energy distribution and cannot directly reflect non-linear temporal characteristics or higher-order interactions between different textures (Jamali and Sammut, 2010); instead, the tactile system must integrate signals across the entire sensing array. Our ResSE-CNN-LSTM model addresses this by utilizing convolutional layers to capture localized spatial gradients and LSTM units to track their temporal evolution. The superior performance of this architecture over baseline models suggests that encoding texture spacing is inherently a spatiotemporal integration task, showing spatially non-uniform response patterns similar to the heterogeneous sensing characteristics observed in biological tactile systems.

Furthermore, the efficacy of the ResSE block in enhancing model stability provides insight into robust tactile sensing. In our experimental protocol, participants were allowed to press the texture board with unconstrained force: a condition that typically introduces substantial non-stationary noise. By autonomously recalibrating channel weights, this attention mechanism is conceptually inspired by biological selective attention. When specific regions of the sensing array make poor contact or experience excessive force-induced noise, the model gates these redundant channels by dropping their weights toward 0.0 while prioritizing informative signals. This adaptive weighting explains the 65% reduction in standard deviation (from 14.99 to 5.31%) compared to the baseline LSTM. It demonstrates that the attention mechanism is not merely a performance-boosting feature but a structural necessity for maintaining robustness in unconstrained, real-world environments where perfect contact is rarely achieved. However, it should be noted that ResSE weights still fail to fully explain the contribution of temporal dynamic features. Further analysis requires combining with other interpretability methods (e.g., Grad-CAM or SHAP) to optimize data augmentation strategies and model architecture, thereby enhancing the robust recognition of more complex tactile patterns (Müller-Cleve et al., 2022; Ouyang et al., 2024).

Despite the high overall accuracy, the classification challenge observed between the 5 and 6 mm categories provides a crucial boundary for our current understanding of tactile discrimination limits. At a sliding velocity of 30 mm/s, a 5 mm spacing generates a fundamental frequency of 6 Hz. Notably, the stepper motor used in our setup operates with a characteristic vibration profile that overlaps with this specific frequency range. This frequency-coupling effect suggests that the 32% misclassification rate for 5 mm samples is likely to derive from environmental mechanical interference. Elucidating these environmental artifacts is essential for future iterations of the system. Potential solutions may include implementing adaptive noise cancelation layers or incorporating multimodal feedback loops, such as motor encoder data, into the neural architecture to decouple mechanical noise from the tactile stream.

Several limitations of the present study should be acknowledged. The experiments were conducted using a single sliding velocity, one pressure sensor configuration, regularly spaced textures, and a limited range of texture spacing conditions (3–7 mm). Although these controlled conditions allowed us to investigate the relationship between texture spacing and spatiotemporal tactile responses, they do not fully represent the diversity of real-world tactile interactions. Consequently, the generalization ability of the proposed approach to different sensing hardware, contact conditions, scanning velocities, and more complex natural textures remains to be systematically evaluated. Future work will investigate these factors using a broader range of experimental settings to assess the robustness and scalability of the proposed approach. Meanwhile, although the proposed approach achieved stable recognition performance under naturally varying tactile interaction conditions, contact force variability was not quantitatively characterized in this study. The pressure sensor array recorded spatial pressure responses, while no external force calibration system was used to obtain absolute force distributions. Therefore, the observed robustness should be interpreted within the investigated experimental conditions rather than generalized to arbitrary force variations. Future work will incorporate quantitative force measurement to further analyze the relationship between force variations and tactile recognition performance. Last, Although dominant frequency tracking and harmonic analysis reveal spacing-dependent temporal characteristics at the signal level, the contribution of individual sensor channels to frequency encoding was not systematically investigated in the current study. Future work will analyze channel-specific spectral responses to further characterize the spatial distribution of frequency-related tactile information.

## 5 Conclusion

In this study, we investigated the signal-level representation of periodic structural information in tactile dynamics through an attention-based approach. Our method leverages structural periodicity, texture spacing generates predictable temporal frequency patterns under the tested sliding conditions (*f* = *v*/*d*). Experimental results from SCA intuitively demonstrate that the tactile response in natural states is a spatially heterogeneous process, requiring a high-dimensional decoding scheme. Subsequent ablation and comparative experiments confirm that the proposed ResSE-CNN-LSTM architecture achieves a classification accuracy of 76.80% and provides an overall improvement over traditional SVM and other deep learning baselines. This performance gain is largely attributed to the integration of spatial convolution and temporal memory, which allows the model to decode texture spacing with 1 mm resolution, even under the variability of unconstrained contact forces. Beyond absolute accuracy, the interpretability of the SE channel weights reveals how the model autonomously prioritizes informative sensing regions while suppressing stochastic noise. These findings suggest that incorporating spatiotemporal characteristics of tactile signals may provide a useful computational perspective for future tactile perception studies in robotic and neuro-prosthetic systems, while further validation in these applications is still required. Future research will focus on extending this approach to complex, non-periodic natural textures and integrating the resulting tactile feedback into real-time closed-loop control systems for advanced prosthetic manipulation. Subsequent research is expected to further improve texture spacing resolution while expanding the spacing range, meanwhile combining the algorithm with direct pressure measurement for tactile perception in humanoid robots to achieve a more comprehensive, quantitative, and detailed perception of texture properties. Other potential attempts will be made to encode and decode tactile information based on texture spacings just like Braille. If the spacing of fine textures can be quantified and supplemented with other visual feature information about the target object, it will enable robots to obtain richer information, thus aiding researchers by attaining a more comprehensive understanding of the target object.

## Data Availability

The data analyzed in this study is subject to the following licenses/restrictions: the data that support the findings of this study are available from the corresponding author upon reasonable request. Requests to access these datasets should be directed to tianshi@tju.edu.cn.
